# Activation and lysis of human CD4 cells latently infected with HIV-1

**DOI:** 10.1038/ncomms9447

**Published:** 2015-10-20

**Authors:** Amarendra Pegu, Mangaiarkarasi Asokan, Lan Wu, Keyun Wang, Jason Hataye, Joseph P. Casazza, Xiaoti Guo, Wei Shi, Ivelin Georgiev, Tongqing Zhou, Xuejun Chen, Sijy O'Dell, John-Paul Todd, Peter D. Kwong, Srinivas S. Rao, Zhi-yong Yang, Richard A. Koup, John R. Mascola, Gary J. Nabel

**Affiliations:** 1 Vaccine Research Center National Institute for Allergy and Infectious Diseases, National Institutes of Health, Building 40, Room 4502, MSC-3005 40 Convent Drive, Bethesda, Maryland 20892-3005, USA

## Abstract

The treatment of AIDS with combination antiretroviral therapy (cART) remains lifelong largely because the virus persists in latent reservoirs. Elimination of latently infected cells could therefore reduce treatment duration and facilitate immune reconstitution. Here we report an approach to reduce the viral reservoir by activating dormant viral gene expression and directing T lymphocytes to lyse previously latent, HIV-1-infected cells. An immunomodulatory protein was created that combines the specificity of a HIV-1 broadly neutralizing antibody with that of an antibody to the CD3 component of the T-cell receptor. CD3 engagement by the protein can stimulate T-cell activation that induces proviral gene expression in latently infected T cells. It further stimulates CD8 T-cell effector function and redirects T cells to lyse these previously latent-infected cells through recognition of newly expressed Env. This immunomodulatory protein could potentially help to eliminate latently infected cells and deplete the viral reservoir in HIV-1-infected individuals.

## Abstract (web summary)

The elimination of latently infected cells is a sought after goal in the treatment of HIV-1 infections. Here the authors develop an approach to eliminate latently HIV-1 infected cells by using an immunomodulatory protein, which can activate viral gene expression in these cells and direct T lymphocytes to lyse them *in vitro*.

## Graphical abstract

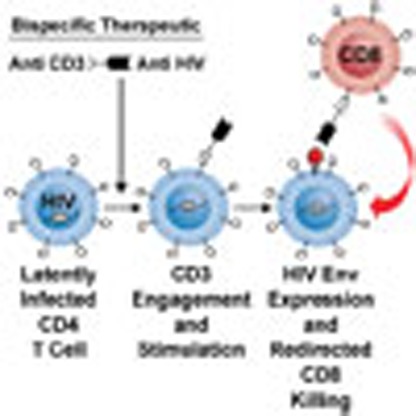

The persistence of latently infected cells during long-term combination antiretroviral therapy (cART) in HIV-1-infected individuals represents a significant hurdle towards a functional cure for HIV-1 (refs 1, 2). Activation and elimination of the latently infected cells in HIV-1 infection has therefore become a major goal of HIV research^3^. A variety of strategies aim to activate HIV gene expression in latently infected cells, which then might be eliminated by antiviral drugs or the immune system (reviewed in ref. 4). The initial use of anti-CD3 and interleukin (IL)-2 treatment to purge the latent HIV-1 reservoir in patients on therapy led to deleterious effects on the immune system and also failed to eliminate the latently infected cells^5^. More recently, the use of histone deacetylase 1 (HDAC1) inhibitors to target latent HIV-1 infection stimulated reactivation of latently infected cells in HIV-1-infected patients; however, the effect in clearing the latent reservoir was modest^6^. Apart from the HDAC1 inhibitors, other molecules such as bryostatin, a protein kinase C activator, and disulfiram have also been shown to activate latent HIV-1 expression^7^,^8^. Although HIV-1 preferentially infects actively replicating cells, it can also infect quiescent cells such as resting CD4^+^ T cells at lower frequencies^9^,^10^. Latent HIV-1 infection of resting memory CD4^+^ T cells is established when activated CD4^+^ T cells return to a quiescent state or through infection of quiescent T cells.

Since most antiretroviral drugs target viral proteins involved in the viral replication cycle, they are unable to eliminate quiescent cells that harbour proviral DNA. During therapy, active viral replication is potently limited by these drugs; however, on treatment interruption, active viral replication resumes in most cases^11^. Consequently, infected individuals must undergo lifelong therapy to limit HIV replication and improve their prognosis. Despite the benefits of cART, treated patients have increased risk for the development of drug-induced diseases including cardiovascular, metabolic and bone disorders^12^,^13^. In addition, there remains a high prevalence of HIV-associated neurocognitive disorders in the cART era^14^. Therefore, eliminating the latently infected cells in HIV-1-infected individuals would limit the dependence on cART drugs for treating HIV-1 infection.

Bispecific antibodies have been designed to redirect T cells for targeting multiple tumours and viral infections^15^,^16^,^17^,^18^,^19^,^20^. While there has been encouraging progress in cancer immunotherapy^21^, progress in eliminating HIV-1 infection has been limited. The lack of efficacy in previous studies was likely because of the use of soluble CD4 as a ligand, which binds with low affinity compared with the aggregated receptors that engage in the immune synapse formed during infection, or the use of anti-HIV-1 antibodies with restricted strain specificity^16^,^17^,^19^, that is, previous bispecific proteins had neither the specificity nor activation potential required to activate and redirect T-cell killing. Recently, combination monoclonal antibody therapy has shown promise in suppressing viral infection in animal models^22^,^23^; however, it does not provide a mechanism for activating infected T cells from latency. The ability of an anti-HIV-1/CD3-bispecific protein to activate and redirect T cells to lyse latently infected T cells provides an immunotherapy that may help to reduce the levels of latently infected cells in HIV-1-infected subjects. Here we have developed a novel immunomodulatory protein by combining the broad recognition of HIV-1 Env (ref. 24) with binding to a T-cell activation glycoprotein, CD3 (ref. 25). This immunomodulatory protein was able to both activate CD4^+^ T cells latently infected with HIV-1 and also redirect CD8^+^ T cells to lyse these infected cells through recognition of HIV-1 Env expressed on these previously latent cells.

## Results

### Production and characterization of immunomodulatory proteins

**Figure 1:**
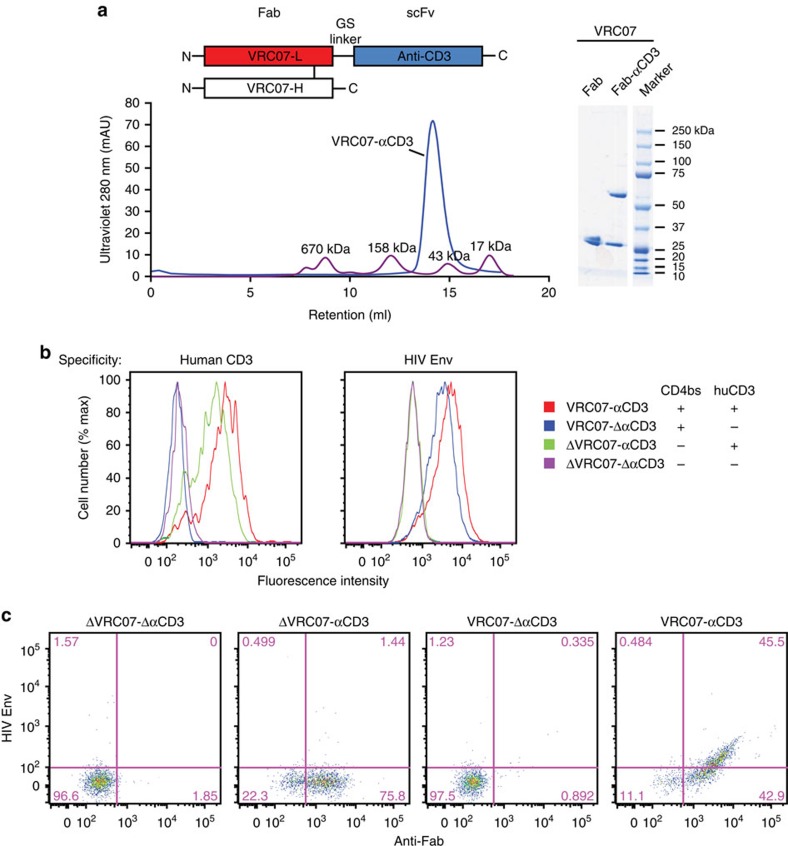
Bispecific immunomodulatory protein binds the CD4-binding site (CD4bs) of HIV Env and CD3. (**a**) Molecular characterization of the bispecific immunomodulatory protein. The chromatogram of VRC07-αCD3 run through a size exclusion column shows the correct molecular size for the bispecific antibody (left). The heavy and light chain fragments of indicated Fab and bispecific antibodies were analysed by reducing SDS–PAGE gel (right). (**b**) VRC07-αCD3 binds to CD3 and HIV-1 Env on the cell surface. Human T cells and HIV-1-infected CEM cells were incubated with bispecific antibodies of the indicated specificities, and bound antibodies were detected by a fluorescein isothiocyanate-conjugated anti-Fab probe. (**c**) The indicated immunodulatory bispecific and control proteins were allowed to bind to naive human T cells and any protein bound to the T cells was detected with flow cytometry after dual staining with fluorescently labelled anti-human Fab and HIV-1 Env (RSC3) probes.

We developed a single immunomodulatory protein by generating a dual specificity antibody that could both activate CD4 cells latently infected with HIV-1 and also facilitate their lysis. The first specificity was directed to the conserved CD4-binding site of HIV-1 Env while the second recognized the CD3 antigen^25^. A bispecific protein was prepared by linking a humanized scFv directed to CD3 to the COOH terminus of the light chain of the Fab region of VRC07 containing a highly active previously described mutation (G54W)^26^ (Fig. 1a). The immunomodulatory protein, VRC07-αCD3, was purified using size exclusion chromatography and showed the expected monomeric molecular weights and composition (Fig. 1a, bottom and right, Supplementary Fig. 1).

Three additional controls were generated that yielded fusion proteins with either specificity alone or a double-negative mutant control. These controls were synthesized either by replacing the VRC07 Fab with an irrelevant control Fab that did not recognize HIV-1 Env, or by substituting the anti-human CD3 scFv with a scFv to rhesus CD3 that did not react with human CD3, or both (Fig. 1b). The VRC07-αCD3 protein demonstrated the expected ability to bind HIV-1 Env (Supplementary Fig. 2) and neutralize HIV-1 (Table 1). It also showed the predicted pattern of binding to CD3 on human T cells and HIV Env on HIV-1-infected CEM-NKr-CCR5 cells using flow cytometry (Fig. 1b, red curve in both panels). The relevant negative control antibodies also displayed the expected non-reactivity with HIV-1 Env and CD3, respectively, using flow cytometry (Fig. 1b, blue versus green versus pink curves in both panels). We also observed concurrent binding using flow cytometry of the VRC07-αCD3 protein to an HIV-1 Env probe, a resurfaced stabilized HIV core protein, RSC3 (ref. 27), when it was bound to naive human T cells through anti-CD3 that was not observed with the relevant single or double mutant control proteins (Fig. 1c).

### Activation of T cells by immunomodulatory proteins

**Figure 2:**
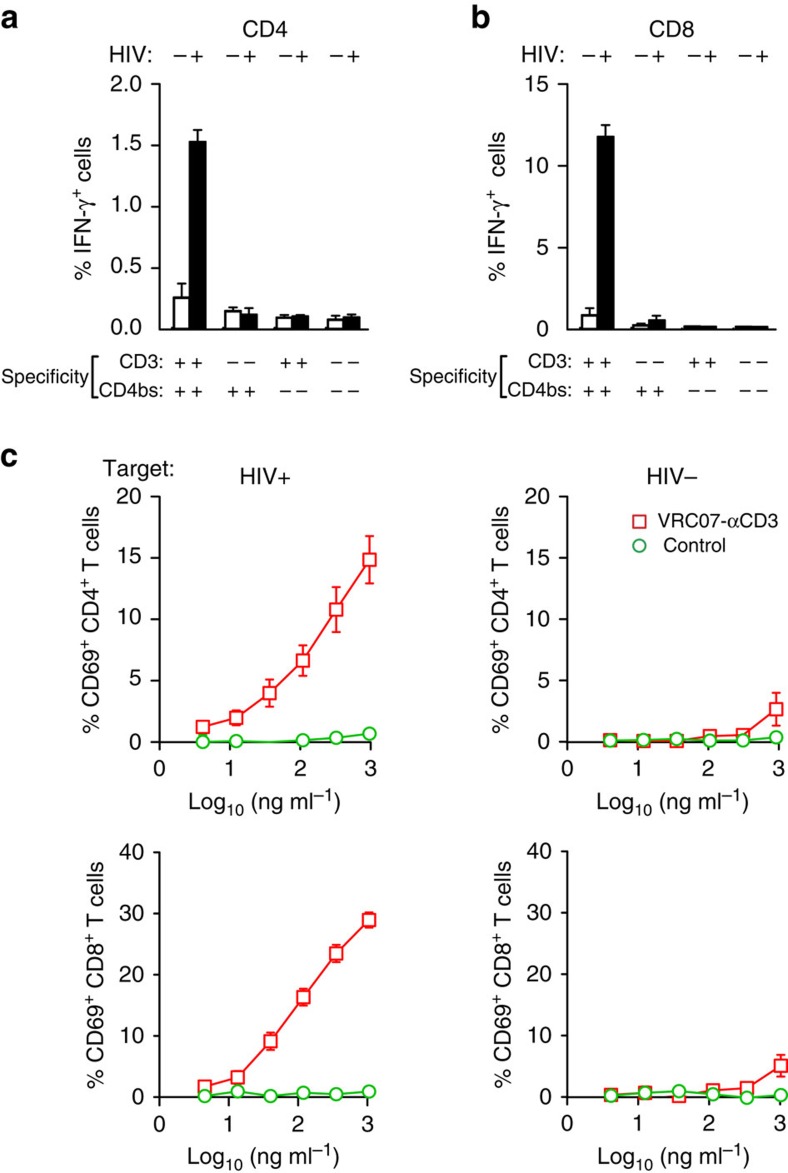
Activation of T cells by an immunomodulatory protein targeting the CD4bs of HIV Env and CD3. (**a**) CD4^+^ and (**b**) CD8^+^ T cells are specifically activated by the bispecific protein. Enriched human T cells were co-cultured with either uninfected or HIV-infected CEM cells (indicated by − or + at the top of each column) in the presence of the indicated bispecific proteins (0.5 μg ml^−1^) and Brefeldin A overnight. The T cells were then stained with an antibody against IFN-γ, and the percentage of T cells expressing IFN-γ was measured using flow cytometry. Representative data from three independent experiments are shown, with each experiment performed with three technical replicates. The error bar represents the s.e. (**c**) Effector cells comprising PBMCs were co-cultured with or without target cells that were either uninfected or HIV-infected CEM cells (indicated by HIV− or HIV+ at the top of each column) in the presence of increasing concentrations of the indicated bispecific proteins and Brefeldin A overnight. The T cells were then stained with an antibody against CD69, and the percentage of T cells expressing CD69 was measured using flow cytometry. The mean of the values at each concentration from three naive donors are shown, with each experiment performed with three technical replicates. Error bars indicate the s.e. at each concentration.

To evaluate whether these immunomodulatory proteins could stimulate the appropriate activation response in T cells after exposure to HIV-1, we analysed the response of human CD4 and CD8 cells in the presence or absence of HIV-1 Env with the VRC07-αCD3 protein and each of the specificity controls. In the presence of HIV-1, CD4 T cells showed a ninefold increase in interferon (IFN)-γ intracellular cytokine staining compared with 1.4-fold in the absence of HIV-1 relative to unstimulated CD4 T cells (Fig. 2a). In contrast, the fusion proteins that reacted with either CD3 or Env alone, or the double-negative control, showed minimal effects (Fig. 2a). Examination of the CD8 response revealed a similar trend in IFN-γ intracellular cytokine staining, with a higher percentage of reactive cells in this population (Fig. 2b). The other control proteins that reacted with Env alone or the double-negative control that was not reactive with CD3 or Env also produced minimal activation. In addition, VRC07-αCD3 stimulated a dose-dependent increase in CD69 expression in CD4 T cells from three different donors in the presence but not the absence of Env (Fig. 2c). In contrast, a control fusion protein that reacted with CD3 alone showed minimal stimulation (Fig. 2c). Examination of the CD8 response revealed a similar pattern of stimulation, with a higher percentage of reactive cells in this population (Fig. 2c). Similar trends were observed for the cytokines IFN-γ and tumour-necrosis factor (TNF)-α, both in CD4 and CD8 T cells (Supplementary Figs 3 and 4). These analyses document that stimulation by VRC07-αCD3 is antigen-specific, and it causes minimal antigen-independent immune stimulation *in vitro*.

### Activation and lysis of latently infected cells

**Figure 3:**
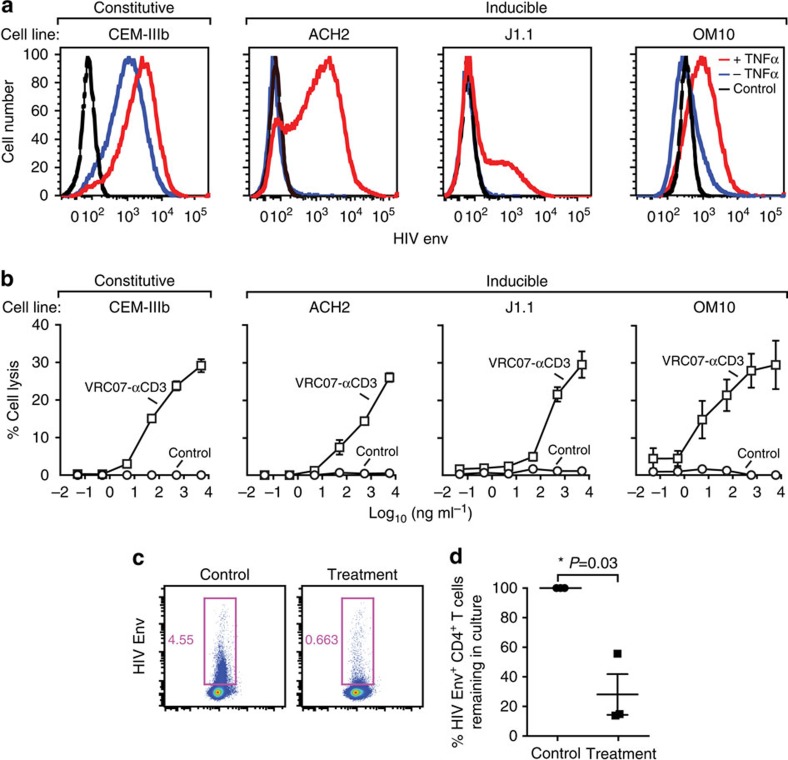
Activation and targeted lysis of chronic and latent HIV-infected cells by VRC07-αCD3. (**a**) Induction of HIV in latent cell lines. Latent cell lines (ACH2, J1.1 and OM10) and a chronic cell line (CEM-IIIb) were cultured in the absence or presence of TNF-α for 14– 16 h and the expression of HIV Env on the cell surface using an allophycocyanin (APC)-conjugated 2G12 was measured using flow cytometry. The increase in the expression of HIV Env indicates the inducible expression in the latent cell lines compared with the constitutive expression in the chronic cell line. (**b**) Targeted lysis of HIV-infected cell lines by VRC07-αCD3. The indicated chronic and latent HIV-infected cell lines were co-cultured with enriched human T cells in the presence of increasing concentrations of VRC07-αCD3 or the indicated mutant control proteins for 14–16 h and per cent lysis of the infected cell line was measured using flow cytometry after staining with a live/dead cell marker. All three monospecific or double-negative controls gave similar results and therefore only one control (double negative) antibody is shown. Representative data from three independent experiments are shown, with each experiment performed with three technical replicates. Error bars indicate the s.e. at each concentration. (**c**,**d**) Reduction in the number of latently infected primary CD4^+^ T cells. Resting CD4^+^ T cells were enriched from PBMCs and infected with HIV-1 BaL after culture in the presence of CCL19 for 3 days. These CD4^+^ T cells were then co-cultured with allogeneic CD8^+^ T cells in the presence of VRC07-αCD3 or the double-negative control protein for 14–16 h. The expression of HIV Env on the surface of CD4^+^ T cells was then measured by flow staining with a fluorescently labelled 2G12 antibody. Representative data from one donor are shown in **c**, and data from three independent donors showing a statistically significant reduction (*P*=0.03, paired two-tailed *t*-test) in HIV Env^+^ CD4 T cells in the presence of the immunomodulatory proteins is plotted in **d**. The mean value is plotted and the error bars represent the s.e.

To determine whether the VRC07-αCD3 protein could stimulate CD8 T-cell lysis of HIV-1-infected cells, we first examined several constitutive or latent yet inducible T leukaemia cell lines. We used CEM-NKr-CCR5 cells chronically infected with HIV-1 IIIb (CEM-IIIb) or latently infected derivatives of ACH2 (ref. 28), Jurkat (J1.1)^29^ and OM10 (ref. 30) cells. CEM-IIIb cells show constitutive expression of HIV-1 Env, in contrast to the three latently infected cell lines that show no detectable expression of HIV-1 Env in the absence of TNF-α (Fig. 3a). When CD8 cells from uninfected donors were incubated with CEM-IIIb cells, they readily lysed the infected target cells in the presence of the VRC07-αCD3 protein that was not seen with the monospecific or double-negative controls. Importantly, all of the latently HIV-1-infected cell lines, grown in the absence of TNF-α, were lysed in a dose-dependent manner by naive donor CD8 cells in the presence of the dual specificity protein but not with the control proteins (Fig. 3b). These findings show that engagement of both targets of VRC07-αCD3 are required to stimulate T-cell cytolysis of target cells latently infected with HIV-1. Next, we asked whether similar activation and antiviral activity could be observed in primary human T cells latently infected with HIV-1. First, we used a model previously shown to mimic latent infection of these cells *in vitro*^9^. Latently infected, CCL19-treated resting CD4^+^ T cells were incubated with syngeneic CD8^+^ T cells in the presence of the VRC07-αCD3 protein or with the double-negative control. A 28-fold reduction in the expression of HIV Env on the surface of the CD4^+^ T cells was observed compared with the control (Fig. 3c,d; *P*=0.03, Student's *t*-test).

**Figure 4:**
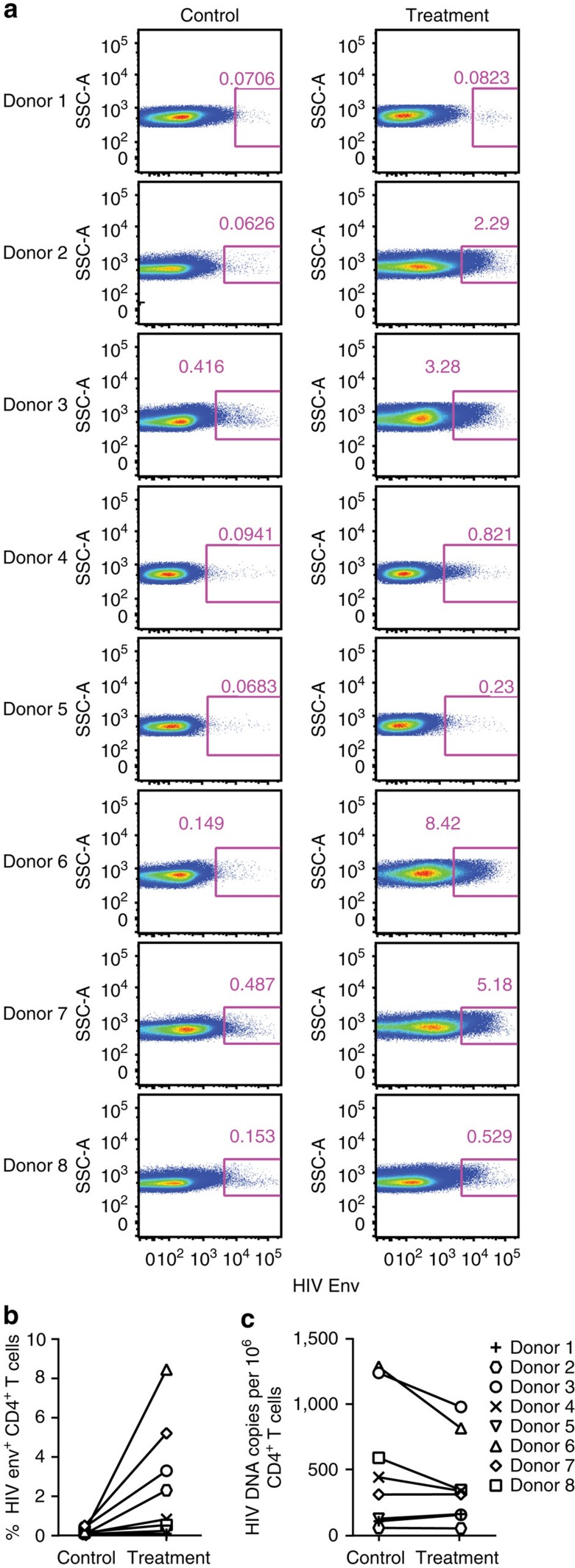
Activation and reduction in the number of latently infected CD4^+^ T cells *ex vivo*. PBMCs obtained from eight HIV-1-infected donors on ART were incubated with the VRC07-αCD3 (Treatment) or a control-bispecific protein that has an active anti-CD3 arm but lacks active VRC07 Fab binding (Control) for 2 days. (**a**) The expression of cell surface HIV Env on live CD4^+^ T cells was detected on day 2 by flow cytometry using an APC-conjugated PGT121 antibody, and the plots for expression of HIV Env on live CD4^+^ T cells for all eight donors are shown. (**b**) The surface expression of HIV Env in control versus treatment groups on day 2 are plotted for each donor. The levels were normalized to the total percentage of CD4^+^ T lymphocytes in the live CD3^+^ T lymphocyte population as determined using flow cytometric analysis. (**c**) The live CD4^+^ T cells on day 2 were also sorted by FACS and the levels of HIV gag DNA in this population was quantitated by real-time PCR. The levels of HIV gag DNA in control versus treatment groups are plotted for each donor after normalization for cell numbers by detection of a housekeeping gene (albumin) in the real-time PCR assay. Because each assay is standardized separately, comparisons can be made within each group but not between them, for example, they do not reflect the per cent of Env^+^ cells relative to those containing proviral DNA.

To confirm the activity of VRC07-αCD3 on latently infected T cells from diverse human subjects, peripheral blood mononuclear cells (PBMCs) from eight antiretroviral therapy (ART)-treated donors (Supplementary Table 1) were treated with the VRC07-αCD3 protein or a negative control-bispecific protein with anti-CD3 recognition but lacking VRC07 binding for 2 days without the addition of any other cytokines or stimuli. This control-bispecific antibody was chosen to demonstrate that the anti-human CD3 scFv does not activate latent cells from HIV-1-infected donors to the same extent as the same protein containing an active VRC07 Fab arm. After the 2-day incubation, live CD4^+^ T cells were sorted and assessed for the frequency of proviral HIV-1 gag DNA using real-time PCR after normalization for total cell numbers in the sorted cell populations. The expression of HIV Env on the surface of live CD4^+^ T cells after the 2-day incubation period was also assessed with flow cytometry using a fluorescently conjugated glycan-V3 targeting broadly neutralizing antibody, PGT121, and expressed as a percentage of the total CD4^+^ T-cell population. These data show that incubation with the VRC07-αCD3 protein, but not the negative control protein, induced Env in CD4^+^ T cells from most donors (Fig. 4a,b). This treatment also led to a decrease in the number of proviral DNA-expressing CD4^+^ T cells in most donors, with no significant increases seen in any donor (Fig. 4c). The ability of the assay to detect changes was limited in donors in whom the frequency of proviral DNA-expressing cells was low; however, these changes were clearly evident in subjects with higher proviral burdens. Collectively, these data demonstrate that the VRC07-αCD3-bispecific protein activates HIV gene expression in latently infected cells and targets them for elimination by redirected CD8 cells.

### *In vivo* safety study with immunomodulatory proteins

**Figure 5:**
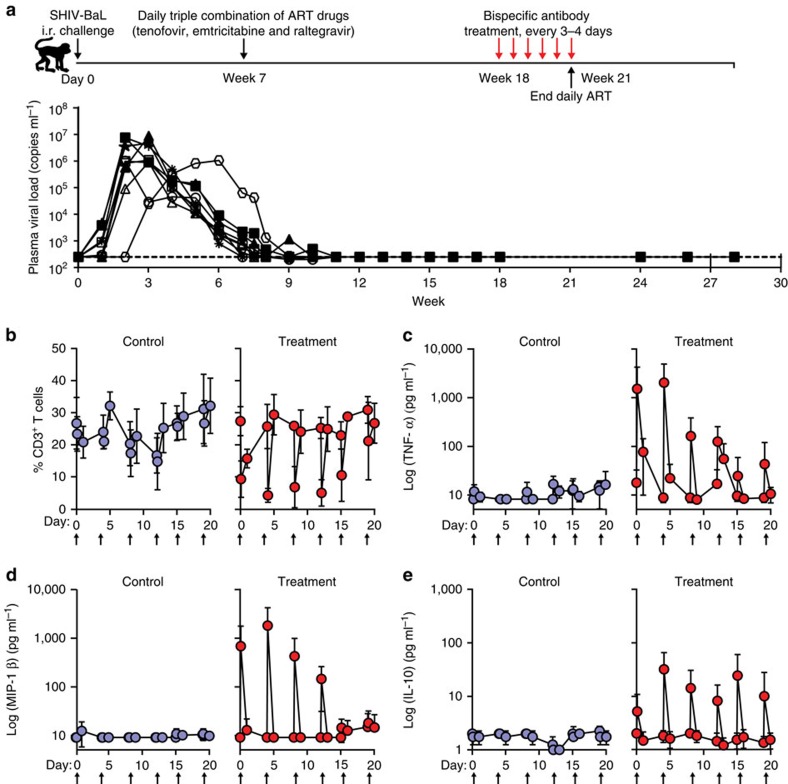
Treatment of SHIV-BaLP4-infected animals with a bispecific immunomodulatory protein. (**a**) Top, experimental schema for the bispecific immunomodulatory protein treatment in which naive rhesus macaques were challenged with SHIV-BaL intrarectally on day 0. At week 7, daily ART was initiated, and the bispecific immunomodulatory or control protein (25 μg kg^−1^), either VRC07-α-rhesusCD3 (treatment, *n*=5) or a control-bispecific antibody that does not bind to either HIV env or rhesus CD3 (control, *n*=4), respectively, were administered every 3–4 days starting at week 18 for a total of six doses. (**a**) Bottom, plasma viraemia in SHIV-BaLP4-infected rhesus macaques that were treated with daily ART beginning on week 7 followed by bispecific immunomodulatory protein treatment on week 18. (**b**) A rapid reversible decline in CD3^+^ T cells was detected in the treatment group 1 h after infusion, which returned to normal levels by 24 h. Infusions are indicated by arrows and the decline is less prominent after infusion 5. Increased levels of TNF-α (**c**), MIP-1β (**d**) and IL-10 (**e**) were detected transiently in VRC07-α-rhesusCD3-treated animals (right) 1 h after infusion during the first four doses, which was less remarkable after day 14. Values represent means±s.e.'s.

To evaluate the safety of the immunomodulatory protein *in vivo*, an analogous VRC07-based bispecific directed to rhesus CD3 (VRC07-α-rhesusCD3) was administered to rhesus macaques infected with SHIV-BaLP4. Antiretroviral therapy-treated SHIV-BaL-infected rhesus macaques were administered a 25 μg kg^−1^ dose of either VRC07-α-rhesusCD3 (treatment, *n*=5) or a negative control-bispecific antibody (Control, *n*=4) at 3–4-day intervals for a total of six doses (Fig. 5a). The negative control-bispecific antibody utilized a human Fab fragment directed to an irrelevant antigen linked to a human anti-CD3 scFv that did not react with rhesus CD3 and therefore recognizes neither of the bispecific targets specifically. None of the animals experienced clinically evident adverse events. All animals were treated with ART throughout the study to repress viraemia, and plasma SHIV gag RNA was undetectable for three consecutive weeks before the immunomodulatory protein infusion (Fig. 5a).

To determine the *in vivo* biological effects of VRC07-α-rhesusCD3, blood samples were collected at 0, 1 and 24 h after each dose. The frequency of CD3^+^ cells in PBMCs and concentration of various inflammatory cytokines in the plasma were then assessed to evaluate safety of the administration. A significant decline in CD3^+^ cells (from 30 to 5% of total PBMC) was observed 1 h after infusion in VRC07-α-rhesusCD3-treated animals, followed by a rapid rebound to the original level by 24 h (Fig. 5b, right), suggesting a redistribution of T cells targeted by VRC07-α-rhesusCD3. In contrast, despite minor fluctuations, the frequency of CD3+ T cells was unchanged for up to 20 days in control animals (Fig. 5b, left). The stimulation of T cells by VRC07-α-rhesusCD3 was assessed by measuring TNF-α, MIP-1β and IL-10 release in the plasma after each infusion. No detectable alterations in cytokine or chemokine release were detected after infusion of the control protein; however, VRC07-α-rhesusCD3-treated animals produced substantial TNF-α (300-3000, pg ml^−1^), MIP-1β (200–2,000 pg ml^−1^) and IL-10 (10–60 pg ml^−1^) 1 h after infusion; levels returned to pre-treatment levels by 24 h (Fig. 5c–e). These results demonstrated successful and well-tolerated stimulation of monkey T cells *in vivo*, presumably including the cells harbouring latent virus, by the anti-CD3 arm of the immunomodulatory protein. This stimulation may then increase surface expression of HIV Env, which can be engaged by the VRC07 arm of the immunomodulatory protein. Notably, both T-cell depletion and cytokine peaks were less remarkable after dose 4; this blunted effect may be explained by the emergence of immune responses against VRC07-α-rhesusCD3 before administration of the fourth dose in most animals (Supplementary Fig. 5). No significant change in the viral load was detected in either VRC07-α-rhesusCD3 or the control group during the study period (Fig. 5a), demonstrating no adverse effect on viraemia induced by the treatment. Overall, these short-term toxicity studies indicate that the treatment was well tolerated, and no major safety concerns were raised by administration of the immunomodulatory protein.

## Discussion

Here a highly potent broadly neutralizing antibody was linked to an activating anti-human CD3 antibody so that both activities were retained and synergistic. This approach is designed to utilize the CD3-signalling component of the bispecific protein to stimulate Env gene expression. By coupling it directly to the VRC07 antibody arm, it allows more efficient recognition and lysis of cells that express low levels of Env. Such levels of HIV Env are not readily quantitated, and there is evidence that low levels of replication occur even during ART^31^,^32^,^33^,^34^,^35^. Specific physiologic stimulation of HIV gene expression further potentiates activation and expression of Env for T-cell-mediated lysis by this immunomodulatory protein. Since there may be cells expressing Env in HIV-infected subjects, these cells likely provide a nidus for attachment of this immmunomodulatory protein. Once attached, it provides a cell surface that can crosslink CD3 and stimulate signalling that further enhances Env expression, further stimulating the process. In the absence of Env, this activity is minimized because the αCD3 arm is a monomer and less readily triggers T-cell activation; hence the selectivity, although some levels of nonspecific T-cell activation because of monovalent CD3 engagement or nonspecific binding of the bispecific protein to cells cannot be excluded, consistent with the T-cell effects observed in non-human primate (NHP) studies. It is likely that both effects are seen *in vivo*, and together they may in fact provide a greater effect.

While this therapy can be evaluated in animal models, such as bone marrow-liver-thymus (BLT)-humanized mice, this model is limited in its ability to evaluate the treatment of HIV latency. First, engrafted T cells are exposed continuously to xenogeneic mouse antigens never encountered in humans, which leads to a state of chronic hyperstimulation. Second, the antigen specificity/repertoire of engrafted human T cells differs from those educated completely in a human. In addition, the interactions of HIV-1 with different cell types are confounded in chimeric mice in which there is variability in levels of engraftment and the relative proportion of human and murine cells. Therefore, these mouse models are unreliable and potentially misleading with respect to human HIV-1 latency, and further studies in humans will be required to test the proof of concept of this approach. Safety concerns may be better addressed in NHPs, and preliminary evaluations reported here suggest that this treatment is well tolerated and does not increase viral replication in the presence of ARVs. Further clinical studies will be required to evaluate its efficacy in humans and its potential to reduce proviral DNA. We suggest that VRC07-αCD3 be administered to patients with concomitant ART to inhibit new cycles of infection, while it both activates and eliminates the latently infected cells of the reservoir.

Having documented the potential of this approach, it is likely that the efficacy of these proteins can be improved, either by addition of specificities directed to other highly conserved Env domains^24^, modulating CD3 affinity^25^, or including a third specificity directed to T-cell co-stimulatory molecules such as PD-1, CD28 or CD137 (ref. 36). HDAC inhibitors have also shown promise in activating HIV-1 from latency^6^. Bispecific activating and targeting proteins could potentially be used in combination with such drugs to better activate and eliminate such infected cells in reservoirs of latent infection. This approach can therefore contribute to the challenging goal of functional eradication of latent HIV reservoirs that might ameliorate the need for lifelong ARV therapy.

## Methods

### Construction of immunomodulatory protein and controls

The cDNAs for human and simianized versions of VRC07 (G54W; Fab) were PCR-amplified from the IgG vector and assembled to anti-human CD3 or anti-rhesus CD3 scFv, respectively, using overlapping PCR. The scFv fragment of an anti-human CD3 monoclonal antibody was linked by a 16-amino-acid GS linker to the light chain of VRC07, and the VH/CH1 domains of VRC07 (G54W) were kept intact to form the immunomodulatory protein. Anti-human CD3 scFv sequences were synthesized using human-preferred codons (GenScript). The control immunomodulatory proteins based on an anti-VRC01 idiotypic antibody 5B8 were similarly assembled by overlapping PCR. All immunomodulatory proteins were configured as Fab-scFv. Assembled cDNAs were cloned into mammalian expression vector VRC8400 that contains a 3C protease cleavage site and a 6 × his tag at the C terminus.

To produce large quantities of the immunomodulatory proteins, 293F cells were transfected with the different expression vectors using 293Fectin according to the manufacturer's protocol (Life Technologies). Five days post transfection, cell culture supernatant was harvested, filtered and buffer-exchanged to Ni-chromatographic binding buffer (50 mM Tris pH 8.0, 150 mM NaCl). The proteins were initially purified using a Ni-Affinity Chromatography column (GE Healthcare Biosciences), followed by gel filtration using a HiLoad 16/600 Superdex 200 pg column (GE Healthcare Biosciences). Only the monomer fractions were collected for further characterization.

The purified immunomodulatory protein was treated with 3C protease (Novagen) at 37 °C to remove the 6 × his tag, followed by passing the products through a Ni-Affinity Chromatography column for removing the cleaved 6 × his tag. The flow-through was collected, buffer-exchanged to PBS and concentrated. The endotoxin level of all purified proteins was measured, and samples with high levels of endotoxin were passed through an endotoxin-removal column (Hyglos). The endotoxin level in all samples used in the *in vitro* and *in vivo* studies was <1 EU mg^−1^.

### Binding of proteins to soluble and cell surface antigens

For binding to soluble antigen, microtitre plates were coated with a resurfaced HIV envelope core protein (RSC3) overnight at 4 °C. The next day, plates were blocked with 5% BSA and after washing increasing amounts of immunomodulatory proteins or antibodies were allowed to bind to the coated RSC3. Bound proteins were detected with a peroxidase-conjugated anti-human Fab (Jackson Immunoresearch) and tetramethyl benzidine detection (Kirkegaard & Perry Laboratories, Inc.). The binding of the immunomodulatory proteins to CD3 and HIV Env on the cell surface was performed using human (HPB-ALL) T-cell lines and chronically HIV-1-infected CEM-NKr-CCr5 cells (CEM-IIIb), respectively. The cells were incubated with the immunomodulatory proteins (20 μg ml^−1^) for 20 min, and bound proteins were detected with flow cytometry using a fluorescein isothiocyanate-conjugated anti-human Fab (Jackson Immunoresearch).

### Neutralization assay

Neutralization of HIV-1 envelope-pseudotyped viruses by the immunomodulatory proteins and antibodies was measured using TZM-bl target cells using previously described methods^27^. In brief, single round of infection Env pseudoviruses were prepared by co-transfecting 293T cells with an Env expression plasmid containing a full gp160 env gene and an env-deficient HIV-1 backbone plasmid. The entry of these pseudoviruses into TZM-bl cells in the presence of serially diluted immunomodulatory proteins or antibodies was then measured by determining luciferase activity in the cell lysates after a 48-h incubation. Neutralization curves were fit by nonlinear regression using a five-parameter hill slope. The 50% and 80% inhibitory concentrations (IC50 and IC80) were reported as the antibody concentrations required to inhibit infection by 50% and 80%, respectively.

### Activation of T cells by immunomodulatory proteins

PBMCs were enriched from buffy coats obtained from naive donors (NIH Blood Bank) using magnetic beads (Miltenyi Biotec). These cells were co-cultured for 16 h with either uninfected or HIV-1-infected CEM cells in the presence of increasing concentrations of immunomodulatory proteins (1–0.01 μg ml^−1^) and brefeldin A. The cells were then stained for surface expression of T-cell markers (CD3, CD4 and CD8) and activation markers (CD25 and CD69) followed by intracellular staining for cytokines (IFN-γ, TNF-α and IL-2) using fluorescently conjugated antibodies (BD Biosciences, eBioscience, Biolegend). The number of CD4 and CD8 T cells expressing each cytokine or activation marker was determined by running the samples on an LSRII flow cytometer and analysing with the Flowjo software (Treestar).

### Activation and targeted lysis of HIV-infected cell lines

Latent cell lines (ACH2, J1.1 and OM10) were obtained from the NIH AIDS Reagent Program. The activation of these cells was performed by culturing in the presence or absence of TNF-α (10 ng ml^−1^) for 14–16 h. Activation was measured by determining the expression of cell surface HIV envelope protein with flow cytometry using an allophycocyanin-conjugated anti-HIV Env antibody (2G12). The CEM-IIIb, ACH2, J1.1 and OM10 cells were labelled with the membrane dye PKH-26 (Sigma) and used as target cells in a cytotoxicity assay. These labelled target cells were co-cultured for 14–16 h at an E:T ratio of 10:1 with enriched human T cells as effector cells in the presence of increasing amounts of the immunomodulatory proteins. The extent of cell lysis in the target cells was determined by staining with a live/dead cell marker (Life Technologies) and measuring the number of dead cells in the labelled target cell population by running the samples on an LSRII flow cytometer followed by analysis using the Flowjo software (Treestar).

### Latent infection of primary human T cells

Anonymized human PBMCs from normal, healthy donors were obtained through the NIH Clinical Center Department of Transfusion Medicine apheresis programme by automated leukapheresis, and resting CD4^+^ and CD8^+^ T cells were magnetically enriched from them. Signed informed consent from the donors was obtained in accordance with the Declaration of Helsinki and the study was approved by the National Institute of Allergy and Infectious Diseases (NIAID) Institutional Review Board. The CD8^+^ T cells were kept in culture in IL-2 (10 IU ml^−1^)-containing media. The resting CD4^+^ T cells were first cultured in the presence of CCL19 (100 nM) for 3 days. These cells were then infected with HIV BaL (multiplicity of infection=0.1) by spinoculation with centrifugation of the cells with the virus at 1,200 g for 2 h at room temperature. These cells were then washed twice with media and cultured for 3 days in the presence of IL-2 (10 IU ml^−1^). The infected resting CD4^+^ T cells were then co-cultured with allogeneic CD8^+^ T cells in the presence of the immunomodulatory proteins (5 μg ml^−1^) for 14–16 h. The co-cultures were then stained with fluorescently conjugated antibodies against T-cell markers CD3, CD4 and CD8, and HIV envelope (2G12) and gag (KC57, Coulter) proteins, followed by flow cytometric analysis using the Flowjo software (Treestar).

### *Ex vivo* culture of latently infected cells

Human PBMCs were obtained from eight HIV-1-infected donors that were on ART (Supplementary Table 1). Signed informed consent was obtained in accordance with the Declaration of Helsinki and the study was approved by the National Institute of Allergy and Infectious Diseases (NIAID) Institutional Review Board. These PBMCs were incubated with immunomodulatory proteins (5 μg ml^−1^) in the absence of supplementation with other cytokines or growth factors. After 2 days of culture, these PBMCs were stained with fluorescently conjugated antibodies against CD3, CD4, CD8, CD20, CD14, live/dead cell marker (Life Technologies) and an allophycocyanin-conjugated anti-HIV-1 envelope antibody (PGT121 (ref. 37)). The stained PBMCs were then run on a FACS Aria (BD biosciences) and the live CD4^+^ T cells were sorted to quantitate the amount of HIV gag DNA in this population using real-time PCR. Flow cytometric analysis was conducted using the Flowjo software (Treestar).

### Real-time PCR for HIV gag DNA

Sorted live CD4^+^ T cells were lysed using the Proteinase K treatment at 56 °C for 2 h followed by inactivation at 95 °C for 5 min. The lysate was spun at high speed to remove debris and 5 μl of lysate was used in a 25-μl PCR as per the manufacturer's recommendations (SsoAdvanced Universal Probes Supermix, Bio-Rad). HIV Gag and human albumin sequences were amplified separately in single-round PCR using the following primers and probes: 5′-GGTGCGAGAGCGTCAGTATTAAG-3′, 5′-AGCTCCCTGCTTGCCCATA-3′, 5-FAM-AAAATTCGGTTAAGGCCAGGGGGAAAGAA-BHQ1-3′, 5′-TGCATGAGAAAACGCCAGTAA-3′, 5′-ATGGTCGCCTGTTCACCAA-3′ and 5′-FAM-TGACAGAGTCACCAAATGCTGCACAGAA-BHQ1-3′. Quantitation was on the basis of a plasmid standard curve that was previously normalized using specific number of sorted cells.

### Treatment of animals with immunomodulatory proteins

Naive rhesus macaques were intrarectally challenged with SHIV-BaLP4 as before^38^. The infection was confirmed by measuring plasma viraemia weekly by a quantitative real-time RT–PCR and allowed to progress for 7 weeks. Then, a combination of three antiretroviral drugs—tenofovir (20 mg kg^−1^ per day, Gilead Sciences), emtricitabine (30 mg kg^−1^ per day, Gilead Sciences) and raltegravir (100 mg BID, Merck)—was administered to these animals to lower viraemia to undetectable levels. Eleven weeks after the start of ART administration, the immunomodulatory proteins (25 μg kg^−1^) were administered over an hour period to these infected animals every 3–4 days. Whole-blood samples were obtained at different time points from all animals to assay for various immunological and virological parameters. All animal experiments were reviewed and approved by the Animal Care and Use Committee of the Vaccine Research Center, NIAID, NIH, and all animals were housed and cared for in accordance with the local, state, federal and institute policies in an American Association for Accreditation of Laboratory Animal Care-accredited facility at the NIH.

### Measurement of peripheral T-cell levels during treatment

Whole-blood samples were obtained from rhesus macaques at various times during treatment with the immunomodulatory proteins. The whole blood was directly stained with fluorescently conjugated antibodies against CD3, CD4, CD8, CD69 and human Kappa chain to assess the distribution of T cells and detect any bound immunomodulatory proteins on the T cells. After incubation with the antibodies, the blood was then lysed with RBC lysing buffer (BD Biosciences) and run on an LSRII flow cytometer to assess the proportions of peripheral T cells at different times during treatment.

### Quantitation of cytokines and chemokines in the plasma

Cytokines (TNF-α and IL-10) and the βeta (β)-chemokine-MIP-1β were measured simultaneously with either the Milliplex Non-human Primate Cytokine/Chemokine kit (Millipore) or the Fluorokine Multianalyte Profiling kit (R&D) using the Luminex xMAP multiplexed bead system (Millipore), according to the manufacturer's instructions. Results obtained from the Luminex xMAP system were analysed automatically by the Luminex xPONENT software programme (Millipore) using a standard curve derived from recombinant cytokine and chemokine standards.

## Additional information

**Accession codes:** The sequences for the various bispecific constructs have been deposited with Genbank with the accession numbers KT365994-KT366000.

**How to cite this article:** Pegu, A. *et al.* Activation and lysis of human CD4 cells latently infected with HIV-1. *Nat. Commun.* 6:8447 doi: 10.1038/ncomms9447 (2015).

## Supplementary Material

Supplementary Information Supplementary Figures 1-5 and Supplementary Table 1

## Figures and Tables

**Table 1:** Neutralization IC50 titres (μg ml^−1^) for the indicated immunomodulatory bispecific and control proteins against three representative HIV-1 strains from clades A, B and C.

**Virus**	**Clade**	**VRC07 IgG**	**VRC07 Fab**	**VRC07-αCD3**
Q23.17.SG3	A	0.036	0.056	0.025
AC10.29.SG3	B	0.114	0.576	0.346
ZM53.12.SG3	C	0.217	0.524	0.240

## Notes

** Disclaimer:**
