# Supplementary material for: Activation and lysis of human CD4 cells latently infected with HIV-1
Source: Nat Commun. 2015 Oct 20;6:8447. doi: 10.1038/ncomms9447 (PMC4633990; doi:10.1038/ncomms9447)
Supplement: Supplementary Information — Supplementary Figures 1-5 and Supplementary Table 1 [file ncomms9447-s1.pdf]

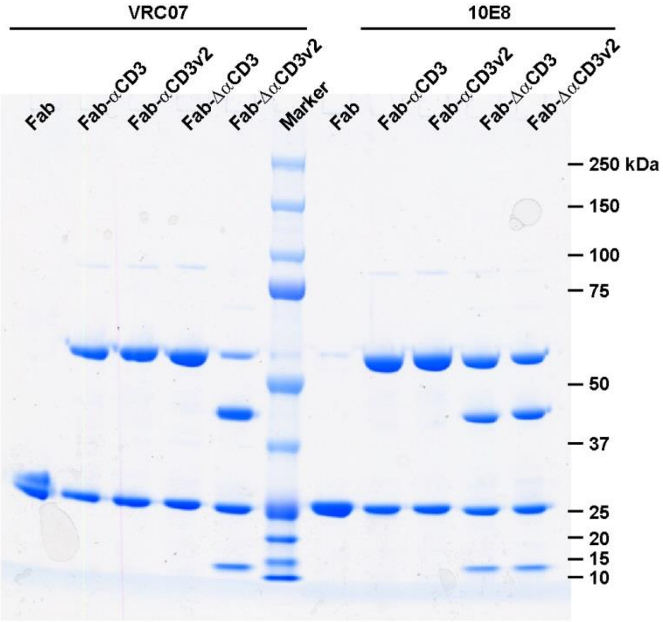

Supplementary Figure 1. SDS-PAGE gel analysis of immunomodulatory proteins. The heavy and light chain fragments of indicated Fab and bispecific proteins were analyzed by reducing SDS-PAGE gel.

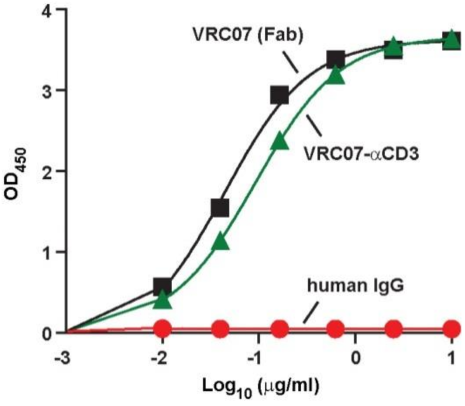

Supplementary Figure 2. VRC07 Fab and bispecific immunomodulatory derivatives bind to HIV Env. The antibodies at increasing five-fold concentrations were allowed to bind to a resurfaced HIV Env fragment containing the CD4bs that was coated on ELISA plates and the bound antibodies were detected using an HRP-conjugated anti-Fab probe.

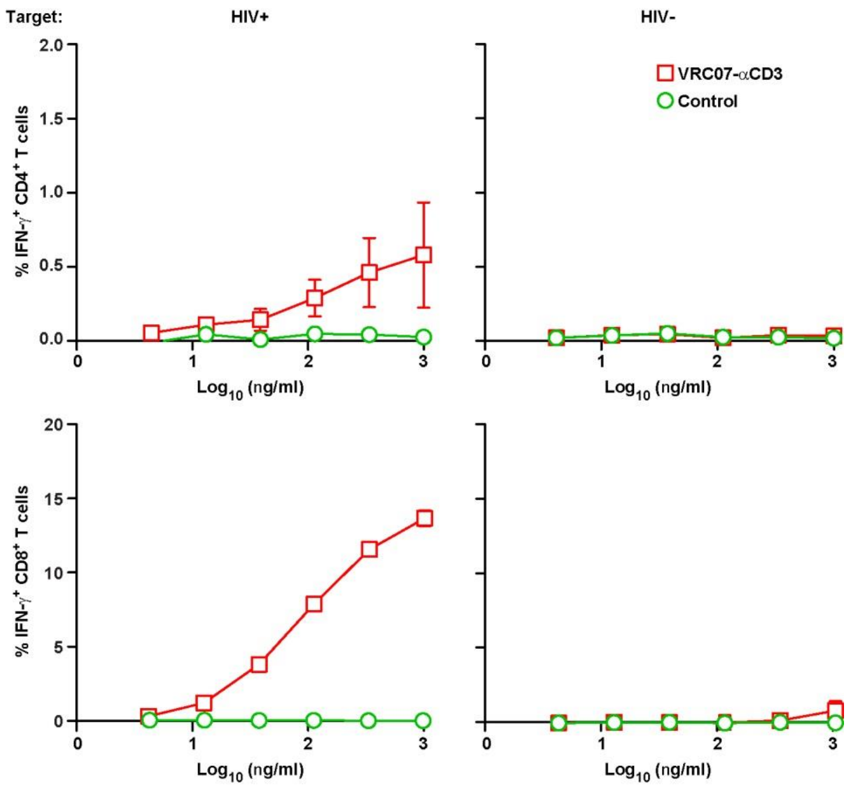

Figure 3. Activation of T cells by a bispecific immunomodulatory protein targeting the CD4bs of HIV Env and CD3. a, $CD4^+$ and **b,** $CD8^+$ T cells are specifically activated by the bispecific protein. Effector cells comprising of peripheral blood mononuclear cells (PBMCs) were co-cultured with or without target cells that were either uninfected or HIV-infected CEM cells (indicated by HIV- or HIV+ at the top of each column) in the presence of Brefeldin A and increasing concentrations of the indicated bispecific proteins overnight. The T cells were then stained with an antibody against IFN-γ and the percentage of T cells expressing IFN-γ was measured by flow cytometry. The mean of the values at each concentration from three naive donors are shown, with each experiment performed with three technical replicates. Error bars indicate the standard error at each concentration.

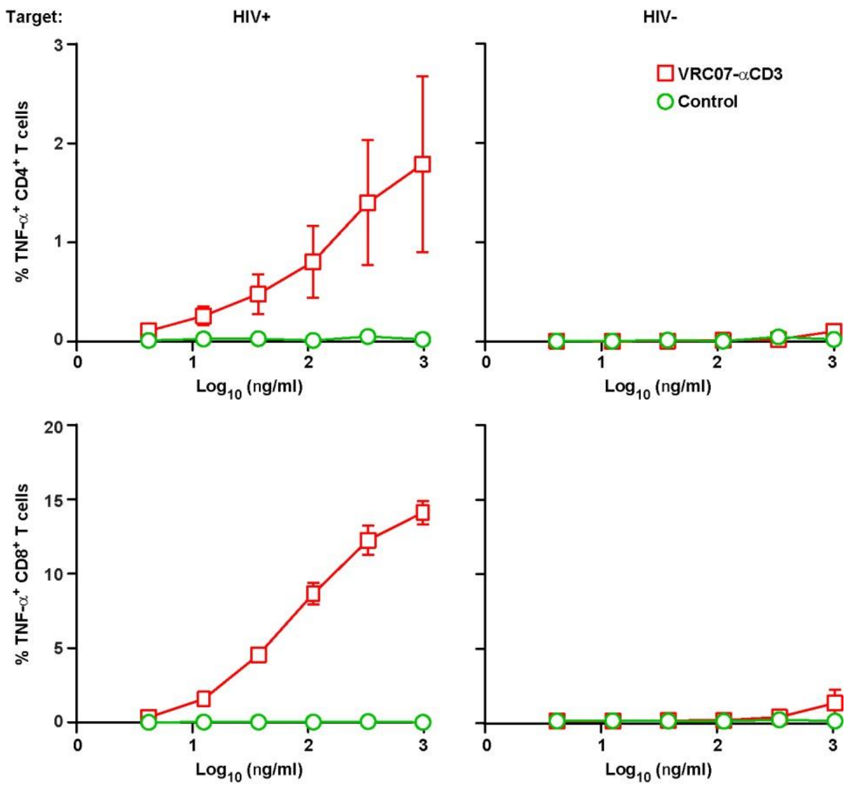

Supplementary Figure 4. Activation of T cells by a bispecific immunomodulatory protein targeting the CD4bs of HIV Env and CD3. a, $CD4^+$ and **b,** $CD8^+$ T cells are specifically activated by the bispecific antibody. Effector cells comprising of peripheral blood mononuclear cells (PBMCs) were co-cultured with or without target cells that were either uninfected or HIV-infected CEM cells (indicated by HIV- or HIV+ at the top of each column) in the presence of Brefeldin A and increasing concentrations of the indicated bispecific proteins overnight. The T cells were then stained with an antibody against TNF-α and the percentage of T cells expressing TNF-α was measured by flow cytometry. The mean of the values at each concentration from three naive donors are shown, with each experiment performed with three technical replicates. Error bars indicate the standard error at each concentration.

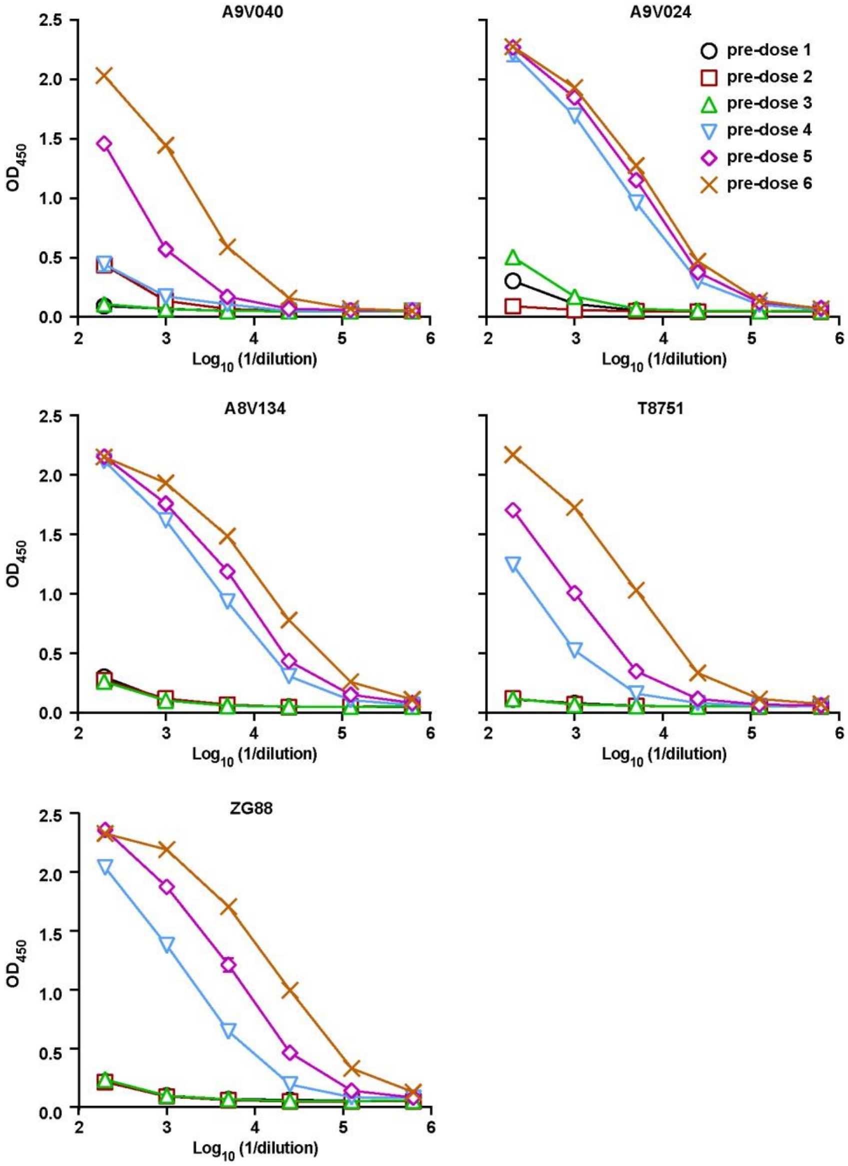

Supplementary Figure 5. Antibody responses against bispecific immunomodulatory protein in nonhuman primates. Serially diluted plasma samples from SHIV-BaLP4 infected rhesus macaques were assessed for binding to VRC07-αrhesusCD3 before each dose of VRC07-αrhesusCD3. Microtiter plates were coated VRC07-αrhesusCD3 and serially diluted plasma from the indicated time point for each animal was then added to the plates. Bound antibodies were detected by a HRP-conjugated anti-monkey IgG. The absorbance at 450 nm (OD450nm) is plotted for each sample.

Supplementary Table 1. HIV-1 infected donors on anti-retroviral therapy

Donor	Year of HIV diagnosis	Year started therapy	ARVs	Day of visit	CD4 T cell count/μl of blood	Plasma viral load (copies/ml)
**1**	1986	2002	Kaletra, Viread, Epivir	6/23/2008	817	<50
**2**	1989	2006	Reyataz, Truvada, Norvir	4/30/2008	959	<50
**3**	1994	1999	Reyataz, Truvada, Norvir	6/2/2008	374	<50
**4**	1995	1997	Atripla	2/20/2007	1222	<50
**5**	1995	1997	Reyataz, Epizicom, Norvir	4/2/2008	769	<50
**6**	1996	2000	Lexiva, Truvada, Norvir	5/14/2008	516	<50
**7**	2006	2006	Kaletra, Ziagen, Truvada	7/1/2008	901	<50
**8**	2006	2007	Atripla	3/25/2008	932	75
